# Comparative study of five-year cervical cancer cause-specific survival prediction models based on SEER data

**DOI:** 10.1038/s41598-025-04540-0

**Published:** 2025-07-02

**Authors:** Yuping Pu, Jundong Liu, Kei Hang Katie Chan

**Affiliations:** 1 Department of Biomedical Sciences, City University of Hong Kong, Kowloon, Hong Kong SAR China; 2 Department of Electrical Engineering, City University of Hong Kong, Kowloon, Hong Kong SAR China; 3 Department of Epidemiology, Center for Global Cardiometabolic Health, Brown University, Providence, RI USA

**Keywords:** Data mining, Machine learning, Cervical cancer

## Abstract

Cervical cancer (CC) is a major cause of mortality in women, with stagnant survival rates, highlighting the need for improved prognostic models. This study aims to develop and compare machine learning models for predicting five-year cause-specific survival (CSS) in CC patients and evaluate their performance against traditional methods like the Cox Proportional Hazards model. Using data from the Surveillance, Epidemiology, and End Results (SEER) program, we applied the Synthetic Minority Over-Sampling Technique to address class imbalance and used stepwise forward selection, feature importance, and permutation importance for feature selection. The Gradient Boosting Survival Analysis (GBSA) model outperformed others with an Inverse Probability of Censoring Weighted Concordance Index of 0.835 and an Integrated Brier Score of 0.120. SHAP value analysis identified tumor stage and surgical resection as key factors. These findings address a critical gap in CSS prediction for CC patients and offer insights for clinical decision-making and personalized treatment. The GBSA model provides more accurate survival predictions, aiding clinicians in tailoring treatment strategies to improve patient outcomes. However, the retrospective study design, potential SEER data entry errors, and the lack of genetic markers and detailed treatment protocols should be considered when interpreting the results.

## Introduction

According to the 2024 American Cancer Statistics, cervical cancer (CC) has become the third leading cause of cancer death among young women since 2019^1^. CC is predominant in 25 countries and the leading cause of cancer mortality in 37 countries, and the survival trends for CC have plateaued^1,2^. Cause-specific survival (CSS) estimates cancer-specific mortality by excluding deaths from other causes, making it particularly suitable for cancers like CC, where cause of death is primarily attributed to the disease, especially in HPV-related cases. Forjaz de Lacerda et al. (2019)^3^ demonstrated that CSS provides reliable survival estimates in such cases, supporting its use as the primary outcome measure in this study.

Despite the potential of machine learning (ML) in survival analysis^4^, several challenges remain, particularly in CC. These include handling censored survival times, managing data imbalance, and addressing complex, non-linear relationships between demographics, tumor stage, and other treatment variables^5^. These issues complicate the development of accurate and generalized models. To address these issues, methods such as the Survival Tree (ST) are designed to maximize survival differences among patient groups in a binary tree structure^6^. The Random Survival Forest (RSF) extends this approach by constructing multiple survival trees and leveraging their average for improved prediction while mitigating overfitting^7^. Furthermore, Gradient Boosting Survival Analysis (GBSA) uses regression trees to incrementally optimize the negative gradient of the loss function, thereby enhancing model generalizability and reducing overfitting^8^. According to Milad Rahimi et al*.*'s research, ML techniques have demonstrated superior accuracy in predicting survival outcomes for CC patients compared with traditional statistical methods^9^, underscoring the potential of ML for improving prognostic assessments in this context. In addition to ML models, traditional statistical approaches such as Cox Proportional Hazards (CoxPH) and Cox Time-Varying (CoxTV) models were also included for comparison to provide a broader evaluation framework^10^.

CC poses a significant health challenge, and predicting patient survival outcomes is essential for refining treatment strategies and enhancing patient care. Despite their potential, the application of ML algorithms to predict survival in CC patients remains scarce^9^. While prior research underscores the importance of demographic, tumor, and treatment factors in survival prediction^11–13^, a comparative analysis of predictive models, especially their ability to interact complexly with these factors, is lacking. To address this, we utilized the (Patient, Intervention, Comparison, Outcome, Study Design) PICOS framework to systematically identify relevant studies and refine our research question^14^. In our study’s initial phase, we used the PICOS on PubMed and yielded no results, revealing a notable gap in comparative studies employing ML models to predict CSS in CC patients. This gap provides both a challenge and an opportunity for our study to contribute valuable insights to the field.

Our study aims to compare and analyze five-year CSS prediction models for CC using the SEER database^15^. We address data imbalance via SMOTE and refine feature selection via stepwise forward selection and feature importance methods. Following performance evaluation via fivefold cross-validation, we identified the most effective model and feature subset. Rationality analysis and SHAP value interpretation were conducted to elucidate the model’s predictions^16^. Our work is significant because of its potential to enhance the understanding of CC survival factors, identify optimal predictive models, and contribute to clinical decision-making, thereby optimizing treatment strategies and improving patient outcomes and quality of life.

## Results

### Patient characteristics

**Fig. 1:**
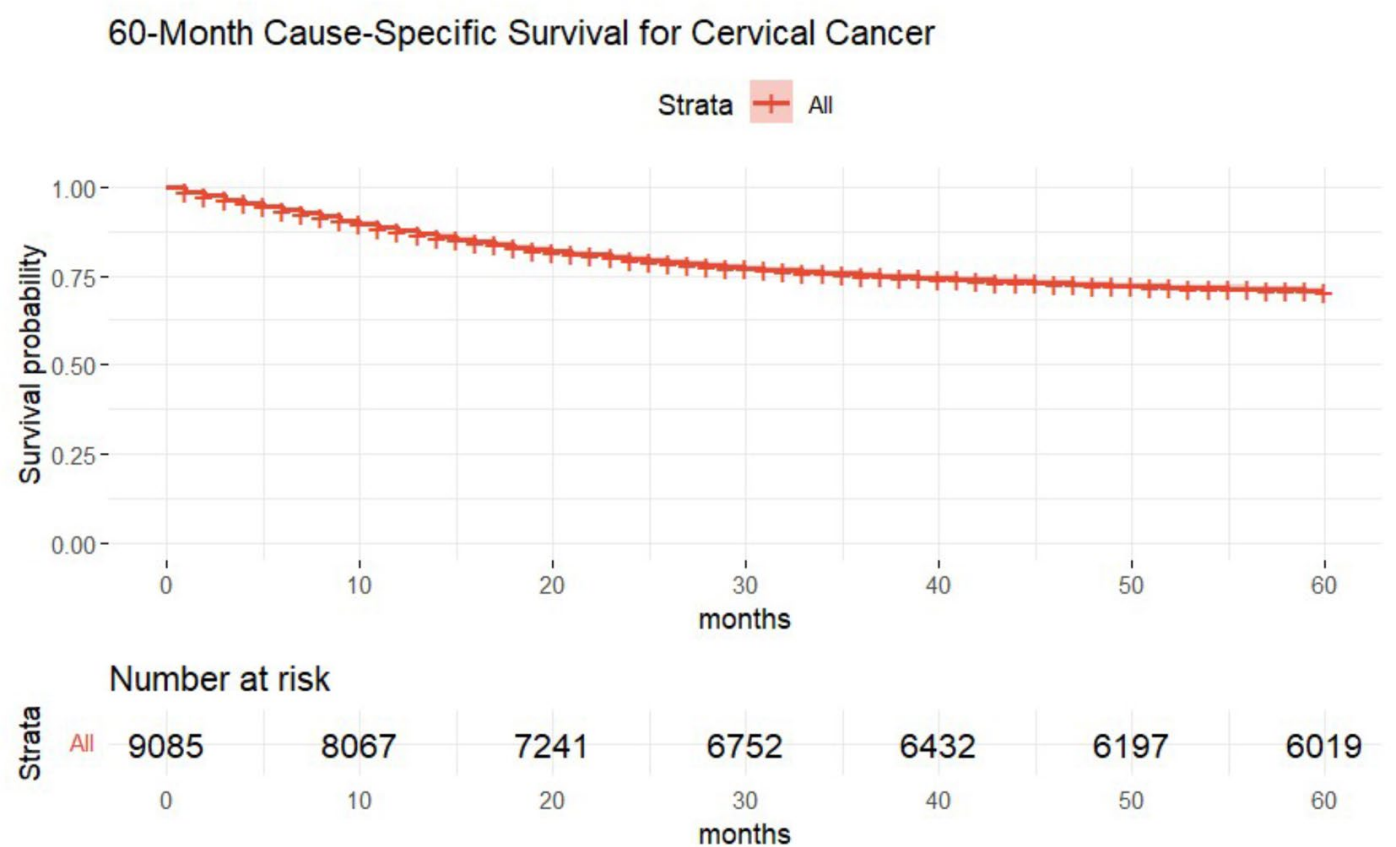
60-Month CSS for Cervical Cancer.

Using the Kaplan–Meier method, we established a survival model for CC patients who met our criteria. In this analysis, we did not stratify the data, meaning that all patients were considered a unified group to assess the overall survival rate of patients with CC. The Kaplan–Meier survival curve depicted in Fig. 1*,* based on truncated data, shows a smooth trend in the CSS rate over 60 months, starting at 100% and declining to approximately 75%, indicating that three-quarters of the patients survived through the study’s observation period. A risk table parallel to the curve details the number of patients at each follow-up milestone, with a gradual reduction reflecting the expected sample size decrease for various reasons, including death and loss to follow-up. We also compared Kaplan–Meier survival curves for non-truncated data to evaluate the impact of survival time discretization. The non-truncated data showed a sharp decline at 60 months due to sparse long-term data (Supplementary Fig S1). Although truncated data may overrepresent survival probabilities at this point, comparing Kaplan–Meier curves revealed no discrepancies in survival trends up to 60 months. Truncating survival times to 60 months ensures consistency in analyzing five-year CSS; therefore, the truncated dataset was used for model analysis to ensure consistency and fairness across comparisons, aligning with the study’s objectives.

We employed a fivefold cross-validation approach to evaluate our model’s predictive performance. (Supplementary Table S1) presents the baseline characteristics of patients across the training and testing sets for each fold, ensuring a balanced validation process. Despite minor statistically significant differences in some folds (*P* < 0.05), these differences were deemed acceptable as the study’s focus lies in the predictive accuracy of the model. To test the PH assumption across different subsets of the data, we performed proportional hazard tests on each of the five folds. The results indicated that across the five folds, the number of features failing the PH assumption test (*P* < 0.05) was as follows: 17 out of 27, 19 out of 27, 18 out of 27, 21 out of 27, and 19 out of 27, respectively (Supplementary Table S2). The consistent pattern of violations across folds confirmed the inapplicability of the Cox PH model for this dataset. Further, it motivated the adoption of ML models that can handle such complexities.

### Feature selection

Our initial dataset comprised 15 original features, excluding ‘Patient ID’, with ‘time’ and ‘event’ designated as dependent variables. After categorizing 13 covariates, we expanded the number of features to 27, which were then reduced to a final set of six through our feature selection process. These features encompass demographics, tumor characteristics, and treatment modalities. The reported subsets were derived through a rigorous, multi-step selection process described in the Materials and Methods section, which integrates stepwise forward selection, RSF-based permutation importance, and GBSA feature importance. The final selected features were validated through cross-fold consistency and are supported by established prognostic knowledge in CC, thereby enhancing the interpretability and clinical relevance of the model. The resulting feature subsets include: (1) FS1, derived through algorithmic selection, which includes “Histology_Squamous_Cell_Carcinoma”, “Radiotherapy_Received”, “Tumor_Stage”, “Chemotherapy_Received”, “Primary_Site_Surgery_Resection”, and “Age”; (2) FS2, consisting of FS1 augmented by the addition of tumor size; (3) FS3, consisting of FS1 with the addition of lymph node surgery status; and (4) FS4, consisting of FS1 with both tumor size and lymph node surgery features included. Regarding the baseline feature subset FS1, although we explained in the Materials and Methods section that normalization was unnecessary, we still applied normalization to further validate this assumption. After applying normalization, the selected feature subsets remained unchanged, indicating that normalization had no impact on feature ranking or selection.

### Model evaluation

**Fig. 2:**
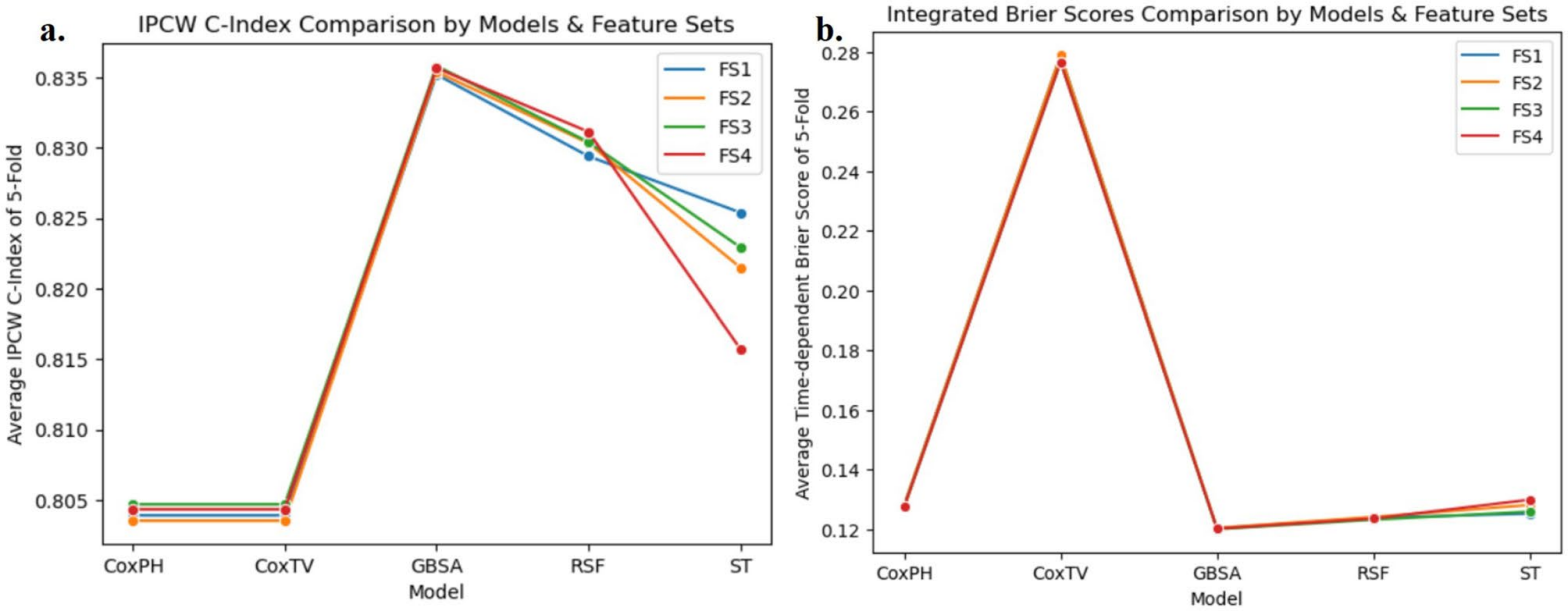
Comparison by Models & Feature Sets. (**a**) IPCW C-index Comparison; (**b**) IBS Comparison.

In our model evaluation, we compared five predictive models: GBSA, RSF, ST, CoxPH, and CoxTV, using three standard survival analysis metrics: IPCW C-index, Integrated Brier Score (IBS), and dynamic area under the ROC curve (dAUC). To ensure robustness, each model was evaluated using fivefold cross-validation across all feature subsets. To assess the incremental predictive value of domain-informed clinical features, we designed an ablation study structured around four feature subsets: FS1 served as the algorithmically derived baseline feature set; FS2 included tumor size; FS3 included lymph node surgery status; and FS4 incorporated both. This design allowed us to evaluate these clinical variables’ individual and combined contributions to model performance. Across all four feature subsets, the GBSA model consistently outperformed the other models. Specifically, for FS1, which represents the baseline set derived through algorithmic feature selection, the GBSA achieved the highest IPCW C-index of 0.835, surpassing RSF (0.829), ST (0.825), CoxPH (0.804), and CoxTV (0.804). This performance trend continued with FS2. The GBSA again achieved an IPCW C-index of 0.835, followed by RSF (0.830), ST (0.821), CoxPH (0.804), and CoxTV (0.804). Figure 2 illustrates the GBSA model’s consistently higher IPCW C-index values and lower IBS across all feature subsets, indicating its robust predictive accuracy. Table 1 shows that all P values from the paired t-tests are less than 0.001 (95% confidence interval), further validating the superior performance of the GBSA model.

**Table 1:** Paired T-test Results of GBSA with RSF, ST, CoxPH, and CoxTV Models.

Metrics	Models	t-value	*P*-value
IPCW C-Index	GBSA—RSF	7.740	< 0.000
IPCW C-Index	GBSA—ST	10.426	< 0.000
IPCW C-Index	GBSA—CoxTV	24.941	< 0.000
IPCW C-Index	GBSA—CoxPH	24.941	< 0.000
IBS	GBSA—RSF	− 10.381	< 0.000
IBS	GBSA—ST	− 11.035	< 0.000
IBS	GBSA—CoxTV	− 478.694	< 0.000
IBS	GBSA—CoxPH	− 21.881	< 0.000

**Fig. 3:**
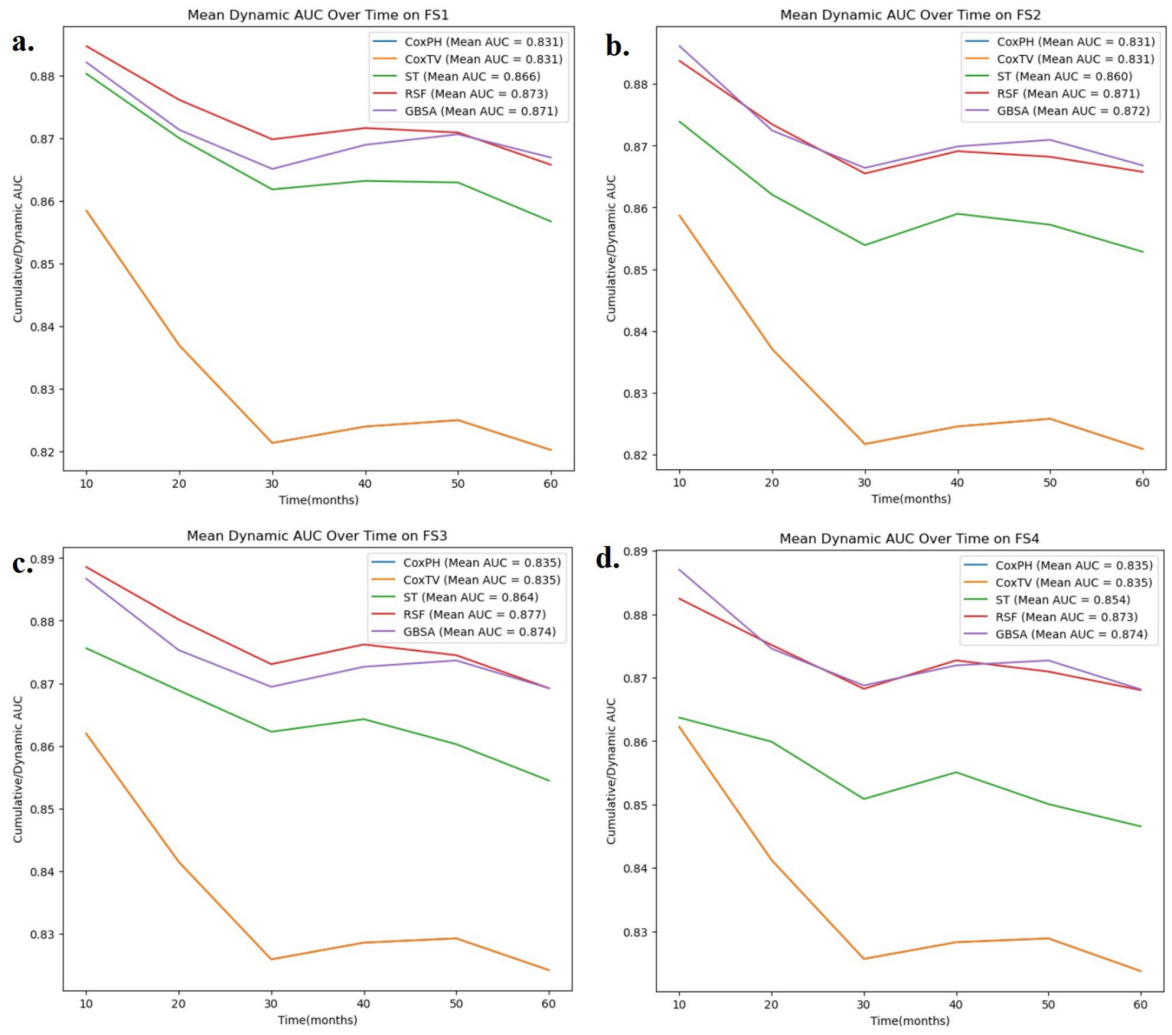
5-Folds Mean Dynamic AUC Over Time by Models & Feature Sets (**a**) FS1, derived through algorithmic selection; (**b**) FS2, consisting of FS1 augmented by the addition of tumor size; (**c**) FS3, consisting of FS1 with the addition of lymph node surgery status; (**d**) FS4, consisting of FS1 with both tumor size and lymph node surgery features included.

Regarding the dAUC, a consistent trend was observed across all four feature subsets, as illustrated in Fig. 3. At the end of the follow-up, the GBSA model maintained the highest dAUC value, followed by the RSF model, and the ST model performed the worst.

We performed a Kruskal–Wallis H test to assess the impact of feature subsets on model performance. The evaluated metrics included the IPCW C-index, IBS, and dAUC. All the Kruskal–Wallis H test results were 3.0, and the *P*-values were 0.392. The test indicated no significant differences among the models’ performances; the results were similar across the different feature subsets, indicating that the selected features contributed equally to model performance.

Using the GBSA model trained with the FS1, we stratified the test set data into low-risk, medium-risk, and high-risk groups on the basis of the 33rd and 66th percentiles of the predicted risk scores. (Supplementary Fig S2) illustrates the distinct survival curves for these groups, with the low-risk group showing the highest survival probability and the high-risk group the lowest. The log-rank test confirmed significant differences in survival times among the groups (*P* < 0.001), validating the model’s stratification effectiveness.

We conducted an additional analysis using a subset of 4694 records with no missing values. The process and the patient characteristics are shown in (Supplementary Fig S3) and (Supplementary Fig S4). The variables we used in this situation were selected by the same algorithm as before; selected features include ‘Chemotherapy_Received’, ‘Radiotherapy_Received’, ‘Primary_Site_Surgery_Resection’, ‘Histology_Squamous_Cell_Carcinoma’, ‘Tumor_Size’, and ‘Tumor_Stage’. The GBSA model achieved an IPCW C-index of 0.811, an IBS of 0.125, and a dAUC of 0.872(Supplementary Fig S5). The GBSA model remained the best-performing model across all evaluation metrics. The result is consistent with the original analysis, demonstrating the robustness of the GBSA model regardless of the dataset size or feature set composition.

Furthermore, we extended the evaluation by assessing model performance without SMOTE handling. Under this preprocessing strategy, the GBSA model achieved the highest IPCW C-index of 0.824 and the lowest IBS of 0.116 among all models. The dAUC for the GBSA model is 0.875, ranking second highest among the models, with the RSF model achieving the highest dAUC value of 0.876(Supplementary Fig S5). The GBSA model exhibited stable performance across different preprocessing strategies.

The comparison across the three data preprocessing strategies shows that the default strategy generally achieved the best or near-best performance in IPCW C-index and IBS across the ML models (GBSA, RSF, ST). Although imputation without SMOTE resulted in slightly lower IBS values and marginally higher dAUC values for some models, the differences were minor and did not outweigh the advantages observed in IPCW C-index. Deletion of cases with missing values resulted in slightly improved performance in traditional models (CoxPH, CoxTV) but generally showed lower performance in comparison to the default strategy, reinforcing that the default strategy is the most effective and robust preprocessing method for this study.

### Feature importance based on SHAP values

**Fig. 4:**
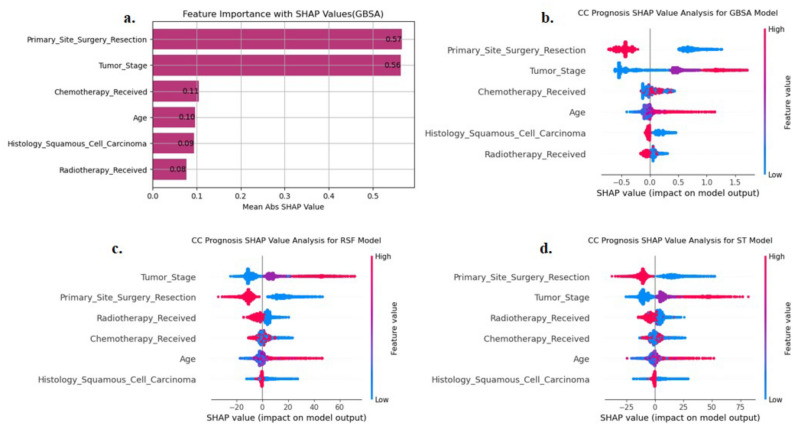
SHAP Explanation. (**a**) Mean Abs SHAP Value of GBSA Model Feature; (**b**) Beeswarm Summary Plots of GBSA Model; (**c**) Beeswarm Summary Plots of RSF Model; (**d**) Beeswarm Summary Plots of ST Model.

The GBSA model trained with the FS1 was selected to evaluate feature importance. Figure 4a ranks individual features by their average SHAP values from the fivefold test set, with the x-axis representing feature importance and longer bars indicating greater influence. ‘Primary_Site_Surgery_Resection’ and ‘Tumor_Stage’ emerged as the most influential features, with SHAP values of 0.57 and 0.56, respectively, underscoring their impact on the model’s predictions. The beeswarm summary plot in Fig. 4b further emphasizes the predominance of ‘Primary_Site_Surgery_Resection’ and ‘Tumor_Stage’, with substantially higher average SHAP values than those of the other features, indicating a strong positive effect on the outcome for patients with CC. However, features such as ‘Histology_Squamous_Cell_Carcinoma’, ‘Chemotherapy_Received’, ‘Age’ and ‘Radiotherapy_Received’ presented more moderate SHAP values, indicating a less pronounced yet notable effect on model output. Consistent with the GBSA model, the RSF and ST models also identified ‘Primary_Site_Surgery_Resection’ and ‘Tumor_Stage’ as the top influential features, highlighting their critical role in predicting five-year CSS in Fig. 4c and d.

### Sensitivity analysis across age subgroups

To evaluate the robustness of the GBSA model across different age strata, we performed a sensitivity analysis by dividing patients into six predefined age groups. Within each age group, the model was trained and validated using fivefold cross-validation with FS1. Performance was assessed using IPCW C-index, IBS, and dAUC. As shown in Supplementary Fig S6-(a), the IPCW C-index generally decreases with increasing age, ranging from 0.862 in the 20–29 group to 0.746 in the 70 + group, despite minor fluctuations across groups. This trend suggests a decline in discriminative performance among older patients. Conversely, IBS increases across age groups, rising from 0.077 in the youngest group to 0.175 in the oldest (Supplementary Fig S6-(b)). The dAUC trajectories, shown in Supplementary Fig S7, exhibit a general downward trend in older cohorts. For the youngest group (20–29 years), the dAUC starts at 0.911 and decreases to 0.882 by the end of the follow-up period. For the 30–39 years group, the dAUC starts at 0.942 and decreases to 0.857. The 40–49 years group exhibits a decrease from 0.916 to 0.858. For the older age groups, the dAUC shows a more pronounced decline, starting at 0.898 for the 50–59 years group and decreasing to 0.863. The 60–69 years group starts at 0.883 and declines to 0.857. The oldest age group (70 + years) exhibits the most noticeable drop, from 0.868 to 0.810.

## Discussion

In our study, we comprehensively evaluated the GBSA, RSF, ST, CoxPH, and CoxTV models via fivefold cross-validation, focusing on the IPCW C-index, IBS, and dAUC as performance metrics. The GBSA model demonstrated superior performance across all three metrics in the FS1, with values of 0.835, 0.120, and 0.872, respectively, suggesting its robustness in survival analysis prediction. The GBSA model outperformed the RSF, ST, CoxPH, and CoxTV models. Violations of the PH assumption significantly hindered the CoxPH model’s performance. The CoxTV model, while capable of handling discrete survival times, exhibited suboptimal performance. Its IPCW C-index was similar to that of CoxPH, but its IBS was consistently higher across folds. These results highlight the superior performance of ML models in handling complex survival data, particularly when the survival time is discretized, and PH assumptions may not hold. We also compared three data preprocessing methods, and the default strategy generally showed the best performance in IPCW C-index and IBS. Our ablation study, which involved sequentially adding clinically meaningful variables to the baseline feature set, revealed no significant performance changes, as confirmed by the Kruskal–Wallis H test (*P* > 0.05). This finding suggests that the feature selection process, which combined both algorithmic and clinically relevant features, is robust and does not overly rely on any single subset of features. However, this similarity also points to a limitation: the possibility of overfitting, as certain features may not be as impactful when applied to different subsets of data. Therefore, while the consistency across subsets in our ablation study supports the reliability of the selected features, it also highlights the need for further evaluation of model generalizability, particularly in external datasets or different patient populations. SHAP value analysis identified ‘Primary_Site_Surgery_Resection’ and ‘Tumor_Stage’ as the most influential features. The higher the degree of tumor spread and the absence of resection surgery at the primary site, the greater the risk of death in the five-year CSS situation for CC patients. These findings have practical implications for clinical decision-making. For example, the analysis suggests that prioritizing surgical intervention for patients with localized tumors or early-stage CC could significantly reduce their risk of mortality. Additionally, identifying patients with advanced tumor stages through SHAP values could guide more aggressive treatment plans, such as chemotherapy or radiation, aimed at improving survival outcomes. Therefore, insights from SHAP values can support personalized treatment strategies and help clinicians focus on high-risk patients who may benefit from more intensive care and interventions. To further assess robustness across demographic cohorts, we conducted a stratified sensitivity analysis by age. The GBSA model attained its strongest predictive performance in the youngest subgroup (20–29 years), achieving the highest IPCW C-index and the lowest IBS among all strata. In contrast, performance declined progressively with age, with the lowest scores observed in the 70 + subgroup. This trend may reflect increasing clinical heterogeneity and comorbidity burden typically associated with aging, which can complicate prognostication. Despite moderate variation across subgroups, the GBSA model preserved strong predictive performance, with IPCW C-index values consistently above 0.74 and dAUC values exceeding 0.81, indicating robust discrimination across all age-defined cohorts.

In past studies, predictive models for survival analysis have often relied on the CoxPH model, which satisfies the PH hypothesis^17^. However, many real-world datasets, including SEER data used in our study, do not meet the PH assumption^18^, limiting the applicability of traditional methods. By employing ML models such as GBSA, RSF, and ST, which do not require the PH assumption, we aimed to address these limitations and provide more robust predictions. These ML models offer greater flexibility in handling complex, nonlinear relationships and censored data. Our study contributes to the gap in the literature by introducing and validating the GBSA, RSF, and ST models for survival analysis without relying on the PH assumption. These models were compared via rigorous fivefold cross-validation, which demonstrated the superior performance of the GBSA model, even without the PH assumption. Notably, our ablation study showed that incorporating additional clinically meaningful variables did not significantly alter the models’ predictive capabilities, suggesting that the baseline feature set was stable and that our overall feature selection process was robust. Furthermore, our analysis identified ‘Primary_Site_Surgery_Resection’^19^ and ‘Tumor_Stage’^20^ as pivotal predictors of survival, which is consistent with previous studies and enhances the model’s accuracy and credibility. These findings reinforce the accuracy and credibility of our model, offering a reliable tool for survival prediction that can be applied to diverse datasets where the PH assumption may not hold.

To further highlight the novelty of our study, we compared our findings with recent studies (Supplementary Table S3). While most prior studies, including Kolasseri and Venkataramana^21^, Yu et al.^22^, Chang et al.^23^, and Rahimi et al.^9^ focused on overall survival (OS) prediction, our study is among the first to systematically explore CSS in CC patients using ML models. Unlike OS, which includes deaths from all causes, CSS isolates deaths specifically attributed to CC, providing a more targeted assessment of treatment efficacy and disease prognosis. For example, Kolasseri and Venkataramana^21^ demonstrated that RSF models outperformed CoxPH models in OS prediction when the proportional hazards assumption was violated. However, our study indicates that GBSA models achieve even higher predictive accuracy, suggesting a superior alternative in such scenarios. Similarly, Yu et al.^22^ utilized ML algorithms for five-year OS prediction in non-metastatic CC patients. However, their study did not incorporate CSS analysis or advanced feature interpretability techniques, such as SHAP value analysis. Chang et al.^23^ applied ML methods to oral cancer OS prediction, focusing on clinicopathologic and genomic markers. Rahimi et al.^9^ reviewed ML models in CC OS prediction but highlighted the lack of studies addressing CSS-specific outcomes. By focusing on CSS and incorporating an ablation study to evaluate clinically relevant variables, our study addresses a critical gap in the literature and provides a more clinically relevant framework for evaluating CC prognosis. Furthermore, our innovative feature selection strategy, combined with robust cross-validation and SHAP-based interpretability, ensures the stability and generalizability of the predictive models while offering actionable insights for clinical decision-making.

We conducted a rigorous multi-index evaluation of the models, utilizing the IPCW C-index, IBS, and dAUC as primary metrics. To support the ablation study, we introduced the Kruskal–Wallis H test to assess the impact of manually added clinical features on model performance. By utilizing the optimal model’s risk stratification method, we graphically represented the prognostic differences among various risk groups. The SHAP value analysis elucidated the model’s predictions, enhancing transparency and offering actionable insights for clinical decision-making, particularly identifying key determinants of five-year CSS in CC patients.

Our comparative analysis of five predictive models based on the SEER database underscores the superiority of the GBSA model for survival prediction. This model’s robustness offers physicians a reliable tool for formulating personalized treatment plans, potentially improving patient outcomes and the quality of care. By providing more precise prognostic estimates, our models can assist medical staff in devising appropriate follow-up plans. Additionally, our emphasis on feature selection methodology, reinforced by the ablation study design, offers a blueprint for future studies and fosters the advancement of survival analysis models. These technical advancements have direct clinical implications. Unlike the CoxPH model, which may not adequately handle complex survival data with PH assumption violations (Supplementary Table S2), the GBSA model provides more robust predictions (IPCW C-index: 0.835 vs. 0.804). This accuracy enables clinicians to stratify patients into distinct risk groups (log-rank *P* < 0.001; Supplementary Fig S2), prioritizing aggressive therapies for high-risk cases (e.g., advanced tumor stages) and reducing interventions for low-risk patients. Furthermore, the model’s identification of key survival factors, including tumor stage (SHAP = 0.56) and surgical resection (SHAP = 0.57, Fig. 4a), directly informs treatment decisions, aligning with guidelines recommending stage-specific management.

Our study, while robust, is not without limitations. There are several limitations in our study. As a retrospective analysis leveraging the SEER database, it is subject to inherent constraints, such as potential data entry errors and incomplete information. In addition, the management of patients with missing data may introduce selection bias. However, our re-analysis of the dataset without missing values and without SMOTE handling consistently demonstrated that the GBSA model outperformed the other models. This consistency suggests that excluding patients with missing data has a minimal impact on the robustness and reliability of our findings. Our study focused on the analysis of CSS, which requires an accurate determination of the cause of death to avoid bias in survival estimates. The absence of personalized treatment details, genetic markers, and clinical test results in the SEER database may limit the comprehensiveness of our risk score interpretations. Given the regional scope of the SEER data and the absence of external validation using real-world clinical datasets, caution is advised when generalizing our findings to broader healthcare settings. Additionally, the ablation study design, while informative, was limited in scope to the evaluation of two manually selected clinical features.

Future research should focus on several key extensions of the current work. First, external validation using real-world clinical datasets is essential to evaluate the model’s generalizability and clinical applicability. Applying the model to hospital-based cohorts can help ensure its robustness across diverse healthcare settings. Second, improving data collection is also critical for enhancing model accuracy. Conducting multicenter studies involving diverse populations and regions would help establish the universality and reliability of the model. Third, incorporating quality-of-life indicators would allow for a more comprehensive evaluation of disease impact and could ultimately improve clinical care by offering deeper insights into patient outcomes. In addition, the model’s ablation study could benefit from a broader selection of clinical variables to enhance interpretability and relevance. These improvements will support more reliable model deployment in varied clinical contexts and facilitate personalized, data-driven treatment strategies.

In conclusion, our study offers several notable strengths that advance CC survival analysis research. First, the systematic comparison of the GBSA, RSF, and ST models via multiple performance metrics and rigorous cross-validation using real-world clinical datasets is essential to evaluate the model’s performance provide a comprehensive benchmark for model evaluation. The GBSA model’s outstanding performance, especially under conditions that violate the proportional hazards(PH) assumption, contributes to predictive modeling in oncology. Second, the robust feature selection process, which combines diverse modeling techniques and a cross-validation framework, ensures the stability and generalizability of the selected features. This methodical approach to feature selection, including the intersection of features from multiple models, highlights our thorough and reliable strategy for identifying key predictors. Finally, applying SHAP values to interpret model predictions adds transparency to our models, facilitating a clearer understanding of the factors influencing five-year CSS in CC patients. The emphasis on feature selection methodology and the superiority of the GBSA model provide a solid foundation for future research and clinical decision-making. Beyond its methodological advancements, our study has broader healthcare policy and clinical practice implications. Enhanced accuracy and interpretability of CSS prediction models enable clinicians to stratify CC patients by risk, allocate resources more effectively, and tailor treatments to individual profiles. For instance, identifying high-risk CC patients through model predictions may support intensive monitoring or early intervention strategies. These contributions pave the way for future research into personalized treatment plans for CC patients.

## Materials and methods

### Research framework

**Fig. 5:**
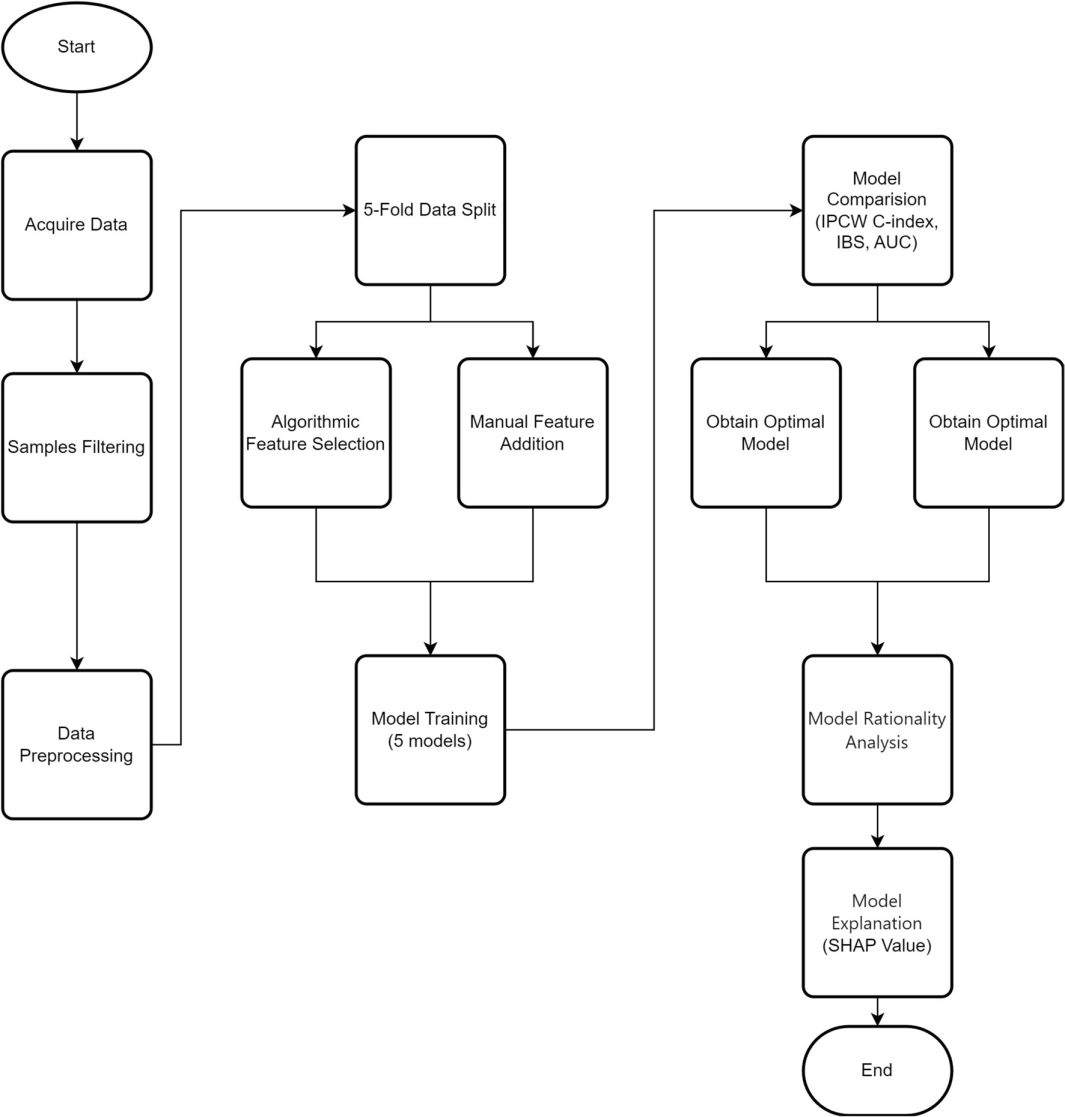
Research Framework.

Our study follows the guidelines outlined in the *BMJ* guide for developing clinical prediction models, published on 3 September 2024^24^. We used the TRIPOD checklist when writing our report^25^.Our study compared different ML models to predict CSS in CC patients and improve the understanding of prognosis via the SHAP value. To this end, we propose a research framework detailed in Fig. 5 to provide more accurate information for clinical decision-making and evaluate the predictive model of CC CSS.

### Data source and selection criteria

**Fig. 6:**
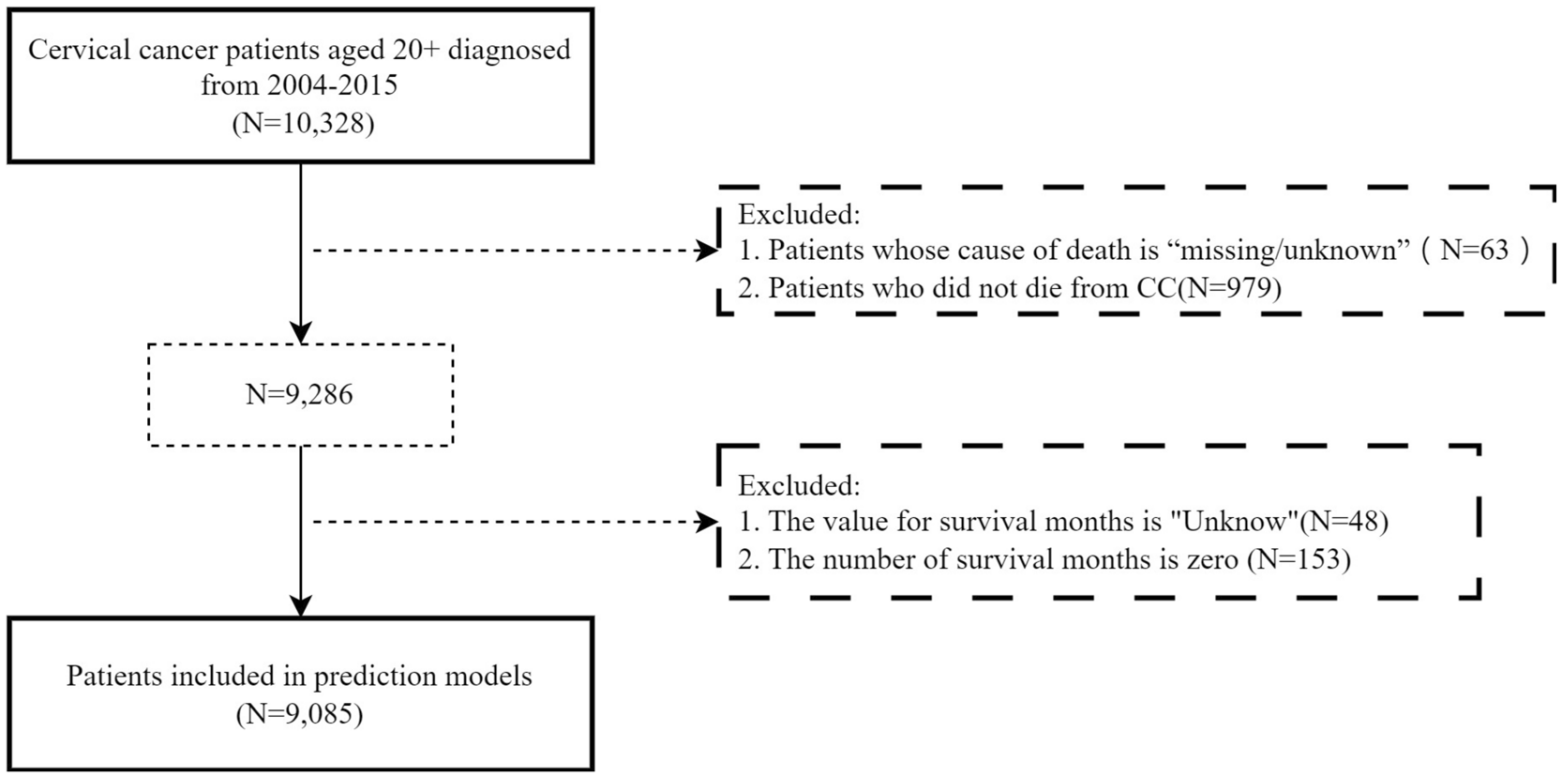
Patient Selection Flowchart.

Data were obtained from the SEER (www.seer.cancer.gov) program of the National Cancer Institute. Using SEER*Stat software (www.seer.cancer.gov/seerstat) version 8.4.3^26^, we accessed the "Incidence—SEER Research Data, 8 Registries, Nov 2023 Sub (1975–2021)" database, which covers approximately 8.3% of the U.S. population (based on 2020 census)^27^. The 8 registries include: San Francisco, Connecticut, Atlanta, Hawaii, Iowa, New Mexico, Seattle, and Utah. The inclusion criterion included female patients aged 20–85 + years who were diagnosed with CC between 2004 and 2015, for a total of 10,328 cases. The exclusion criteria were missing or unknown causes of death (N = 63), noncervical cancer related deaths (N = 979), unknown survival months (N = 48), and zero survival months (N = 153), resulting in a final cohort of 9,085 patients. The patient selection process is detailed in Fig. 6.

### Data selection and preprocessing

Survival times exceeding 60 months were truncated to 60 months to focus on five-year CSS, which aligns with the objective of our study. This truncation ensures consistency in the observation period while reducing the impact of sparse data for long-term survivors. Demographic characteristics, tumor characteristics, and treatment approaches are crucial for CC prognosis^11–13^. Initially, we identified 16 relevant features, which were narrowed to six after preprocessing and feature selection. The selected features included ‘Patient ID’, ‘Marital status at diagnosis’, ‘Age recode with < 1 year olds’ (based on Age at Diagnosis), ‘Race recode (White, Black, Other)’, ‘Primary Site—labeled’, ‘ICD-O-3 Hist/behav, malignant’, ‘Summary stage 2000 (1998–2017)’, ‘Grade Recode (thru 2017)’, ‘RX Summ–Surg Prim Site (1998+)’, ‘RX Summ–Scope Reg LN Sur (2003+)’, ‘Radiation recode’, ‘Chemotherapy recode (yes, no/unk)’, ‘CS tumor size (2004–2015)’, ‘Total number of in situ/malignant tumors for patient’, ‘Survival months’, ‘Vital status recode (study cutoff used)’ and ‘SEER cause-specific death classification’.

Each variable was carefully processed; for example, ‘Unknown’ values were replaced with NA values, and categorical variables with excessive categories were consolidated. We stratified the data into five folds on the basis of the “event” variable. Missing values were imputed via the mode imputation method. Each fold’s test and training sets were filled separately to avoid data leakage. We encoded the ordinal categorical variables and used Python’s “get_dummies” method to handle nominal categorical variables. To address the imbalance of each fold’s “event” variable in the training data, we chose SMOTE because it generates synthetic samples for the minority class, which helps improve model training by providing more representative data^28^. This method has been shown to be effective in handling imbalanced datasets, especially in survival analysis, where the risk of overfitting to the majority class is significant^29^. SMOTE’s ability to balance the dataset allows for more accurate predictions by ensuring that both classes are adequately represented in the training process^29^. To systematically assess the impact of different dataset preprocessing strategies, we compared three approaches: (1) Imputation combined with SMOTE oversampling, which was used as the default preprocessing method for the main analysis; (2) Imputation without SMOTE; and (3) Deletion of cases with missing values. All predictive models were evaluated across these preprocessing strategies using IPCW C-index, IBS, and dAUC metrics. These comparisons were designed to validate the robustness of model performance under different data handling techniques.

### Feature selection

**Fig. 7:**
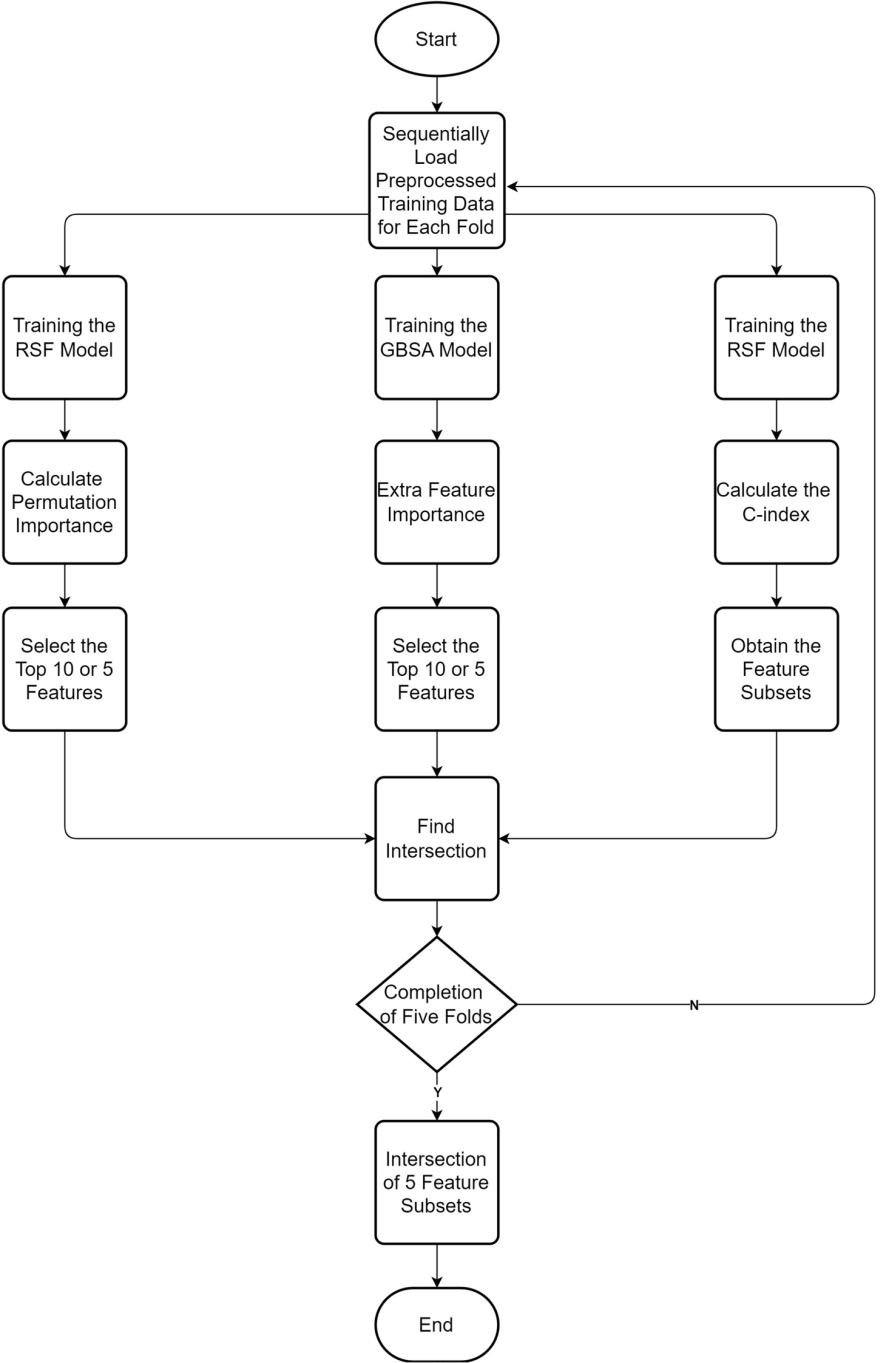
Feature Selection of the Whole Process.

SEER data, while comprehensive, contains a high dimensionality of variables, many of which may introduce noise or redundancy in predictive modeling. To address this, we applied a robust feature selection approach tailored to prioritize clinically relevant variables while optimizing predictive accuracy. Specifically, we engaged three distinct techniques—stepwise forward selection, RSF-based permutation importance, and GBSA feature importance—and derived the final feature subset through their intersection. This method reduces the risk of overfitting and enhances model interpretability. These subsets obtained via these different methods were combined by intersecting to generate a feature subset for each fold. This procedure was repeated for all the folds in the training set. Ultimately, we again intersected the resulting feature subsets from each of the five folds, resulting in the ultimate feature subset. For a clearer grasp of the process, see Fig. 7.

The first and second approaches consider feature importance as a basic criterion for selecting the most important features. However, they differ in how they calculate this importance. The first approach employs an RSF model and assesses feature importance through permutation, observing how much prediction error increases when a feature’s information is randomized. The second method uses a GBSA model to directly calculate feature importance based on its contribution to the model’s performance. The third method used a stepwise forward selection approach based on the C-index derived from the RSF model. The features were incrementally added to the model, and the model’s C-index was evaluated. The process of adding new features stopped when the C-index stopped increasing significantly. The first and second approaches rank features by their importance scores, where the absolute values are irrelevant to the final selection. Specifically, the features are ranked in descending order based on their importance scores. The top features, ranked in descending order of importance, were selected, with a maximum of 10 features chosen based on their non-zero importance. Normalization was therefore unnecessary, as it would not alter the relative rankings. Similarly, the stepwise forward selection approach, which relies on optimizing the C-index, inherently operates without requiring normalization. Consequently, no normalization was applied during any stage of the feature selection process.

### Ablation study

We designed an ablation study to evaluate the individual and combined effects of two domain-informed clinical variables. Based on FS1, which included only algorithmically selected features, we constructed three additional feature subsets: FS2 adds tumor size, FS3 incorporates lymph node surgery status, and FS4 includes both variables. This design allows for a systematic assessment of the incremental predictive value and interpretability provided by clinically significant features. These features were selected based on their critical role in the FIGO 2018 staging system for CC^30^. The FIGO staging system incorporates tumor size and lymph node status to assess the cancer stage, which directly influences treatment decisions and survival outcomes. Tumor size is a well-established prognostic factor, as larger tumors are generally associated with poorer survival outcomes^31^. Similarly, lymph node surgery status provides essential information regarding the extent of cancer spread, which is critical in determining treatment strategies^32–34^. Including these features allows the model to account for important clinical factors that may not be fully captured by the algorithmic feature selection process alone, thereby improving both the interpretability and performance of the model. The inclusion of these clinical variables ensures that the model reflects real-world clinical knowledge, enhancing its practical applicability for healthcare providers. Ultimately, we obtained four feature subsets.

### Predictive models construction

We trained our predictive models on each fold of the training set using the four feature subsets identified earlier. Our comparative analysis included three ML models: ST, RSF, and GBSA, selected for their ability to handle complex, nonlinear relationships in survival data^35^. In addition, we implemented the CoxPH model to provide a benchmark comparison despite our data not meeting the PH assumption^36^. To address this limitation and the discretized survival times in the SEER dataset, we further included the CoxTV model, which accounts for time-dependent covariates. All models were validated using fivefold cross-validation, and performance was evaluated with IPCW C-index, IBS, and other metrics to ensure comparability under identical conditions.

### Model evaluation and explanation

We assessed model performance via three metrics: the IPCW C-index, IBS, and dAUC. The IPCW C-index offers a censoring-adjusted evaluation by using inverse probability of censoring weights to handle censored observations, following the methodology described by Uno et al.^37^. The IBS quantifies prediction error across multiple time points by integrating time-dependent Brier scores, as detailed by Graf et al.^38^. The dAUC measures discrimination performance over time by computing cumulative sensitivity and specificity, based on dynamic time-dependent ROC curves, as described in studies by Hung and Chiang^39^, Lambert and Chevret^40^, and Uno et al^41^. Readers are referred to these studies for detailed formulas and computational procedures. We calculated these metrics for each fold’s test set data and averaged the scores to obtain a final evaluation.

We performed a Kruskal–Wallis H test to assess the impact of feature subsets on model performance. The optimal model, which was selected on the basis of these metrics, was further validated via risk stratification. The risk scores from the test set were averaged, and the samples were categorized into low, medium, and high-risk groups. Distinct survival curves and significant log-rank test results indicate model rationality. SHAP values were used to interpret the model’s predictions, attributing importance to each feature.

### Sensitivity analysis

To assess the robustness of the GBSA model across different patient subpopulations, we conducted a sensitivity analysis by stratifying the dataset into six predefined age groups: 20–29, 30–39, 40–49, 50–59, 60–69, and 70 years or older. For each fold in the fivefold cross-validation scheme, both training and testing sets were split according to these age strata. Within each age group and fold, the model was retrained on the corresponding age-specific training subset and evaluated on the matching test subset. Model performance was assessed using the IPCW C-index, the IBS, and the dAUC. The dAUC was calculated at six evenly spaced time points between 10 and 60 months. These settings were consistent with those used in the primary comparative analysis. This stratified evaluation procedure reflects established practices for assessing model performance and robustness in real-world subpopulations, as supported by prior clinical prediction research^42^.

### Statistical analysis

We conducted statistical analysis on the sample data via R, generated the overall survival curve for the entire sample, and produced baseline tables for both the training and test sets within the fivefold data.

## Supplementary Information

Supplementary Information.

## Data Availability

All data used in this study are publicly available through the SEER database (https://seer.cancer.gov/). Access permissions may vary depending on the user’s institutional affiliation and the applicable data access policies at the time of the request.

## Code availability

The full code is available at the indicated GitHub repository (https://github.com/AngiePu666/SEER_CSS.git).
