# Supplementary material for: Comparative study of five-year cervical cancer cause-specific survival prediction models based on SEER data
Source: Sci Rep. 2025 Jul 2;15:22465. doi: 10.1038/s41598-025-04540-0 (PMC12216411; doi:10.1038/s41598-025-04540-0)
Supplement: Supplementary file 1 — Supplementary Information. [file 41598_2025_4540_MOESM1_ESM.pdf]

Comparative Study of Five-Year Cervical Cancer Cause-Specific Survival Prediction Models Based on SEER Data

Yuping Pu[1], Jundong Liu[1], Kei Hang Katie Chan[1,2,3,*]

1Department of Biomedical Sciences, City University of Hong Kong, Hong Kong SAR, China

2Department of Electrical Engineering, City University of Hong Kong, Hong Kong SAR, China

3Department of Epidemiology, Centre for Global Cardiometabolic Health, Brown University, RI, USA

* Corresponding author, kkhchan@cityu.edu.hk

Supplementary

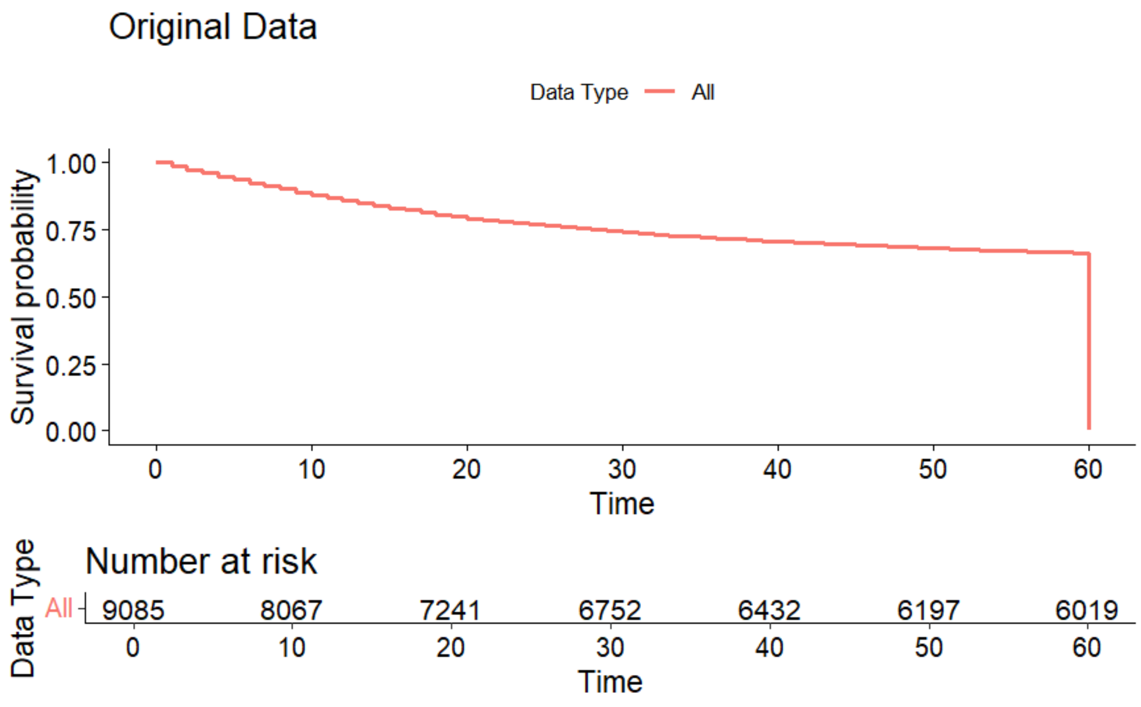

Supplementary Fig S1 60-Month CSS for Cervical Cancer: Untruncated Data

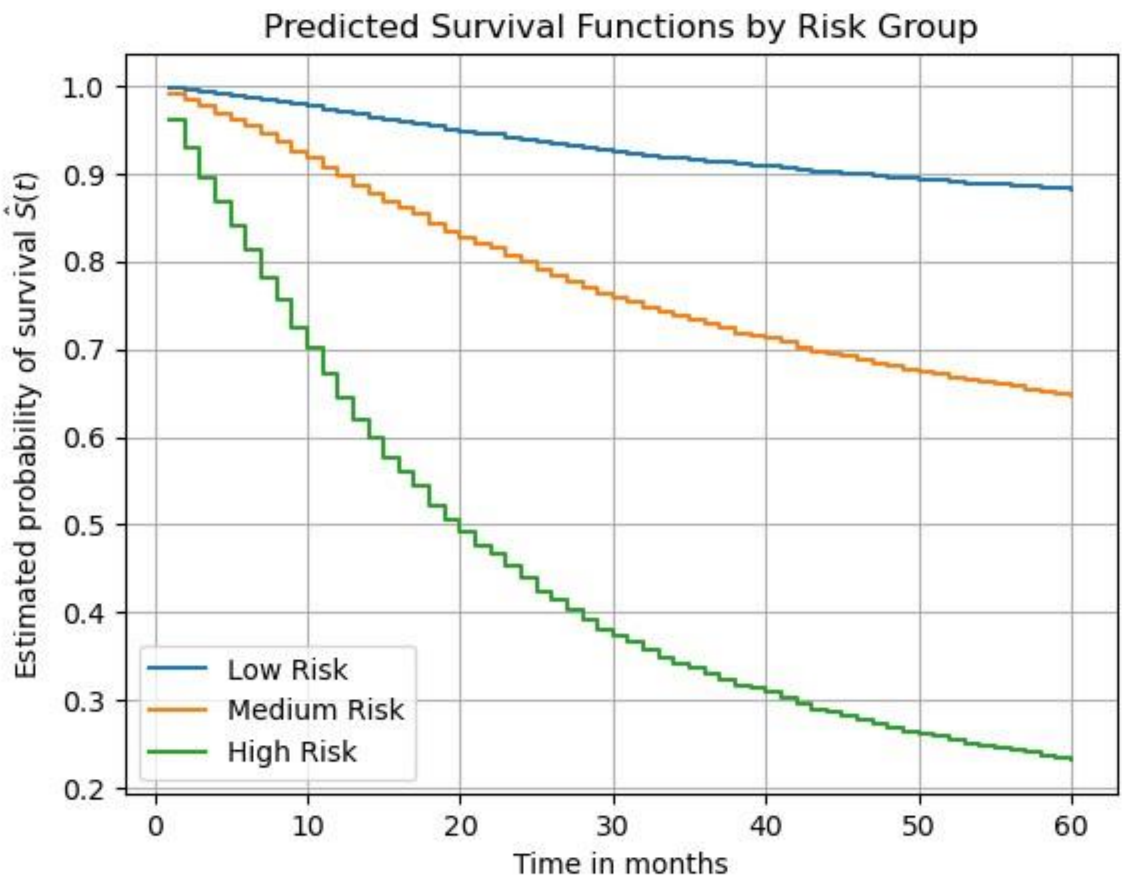

Supplementary Fig S2 Survival Curves by Risk Group Based on GBSA Model

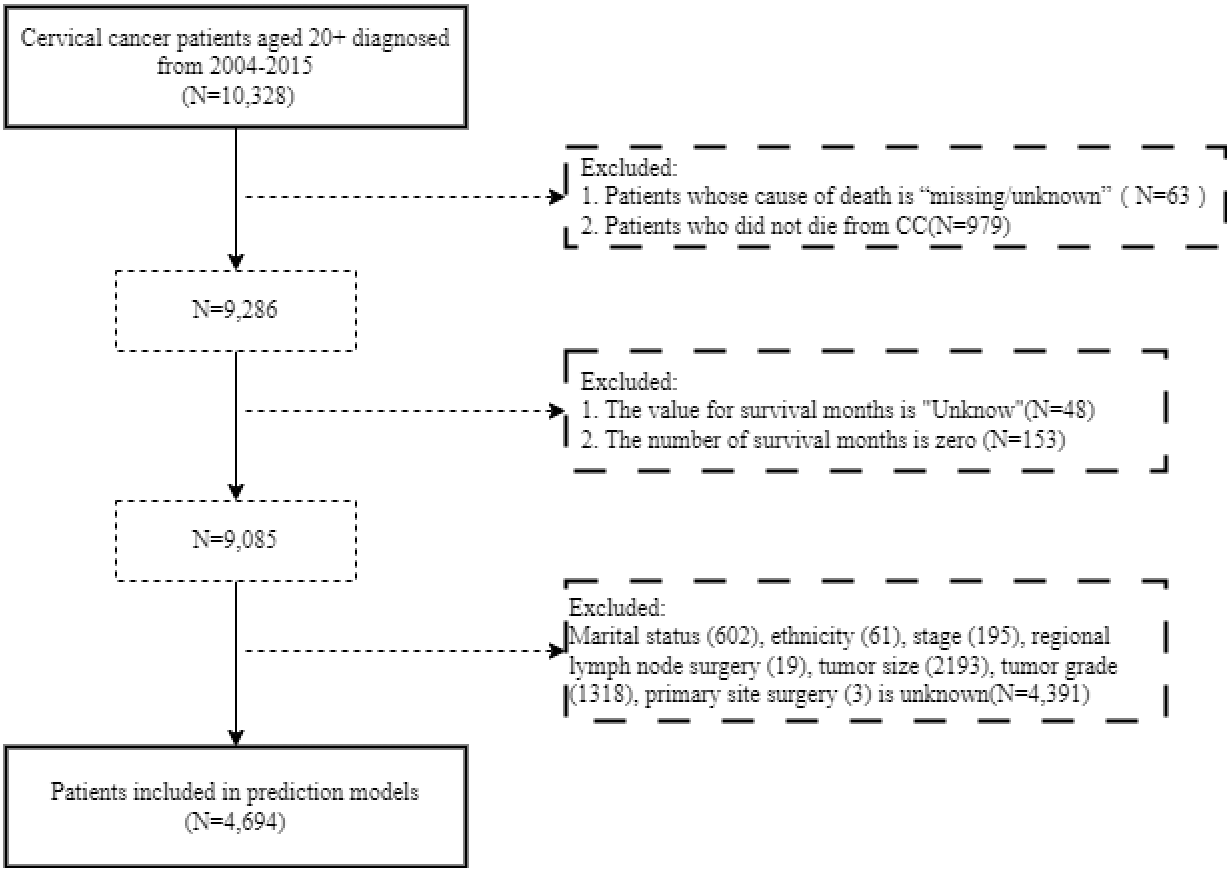

Supplementary Fig S3 Patient Selection Flowchart: No Missing Values Patients

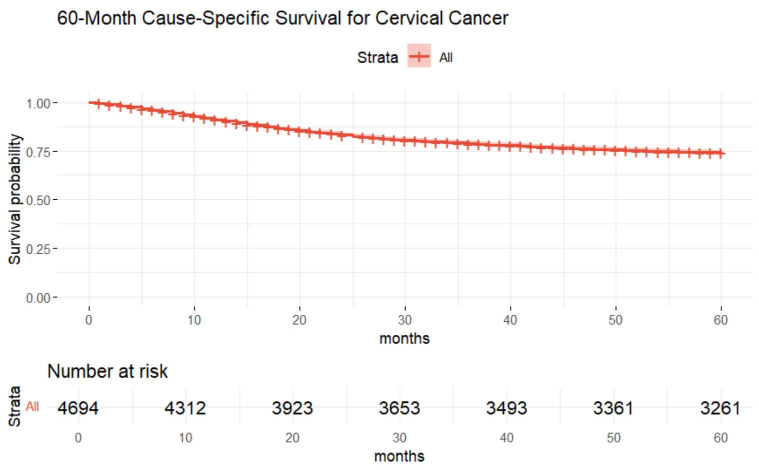

Supplementary Fig S4 60-Month CSS for Cervical Cancer: No Missing Values Patients

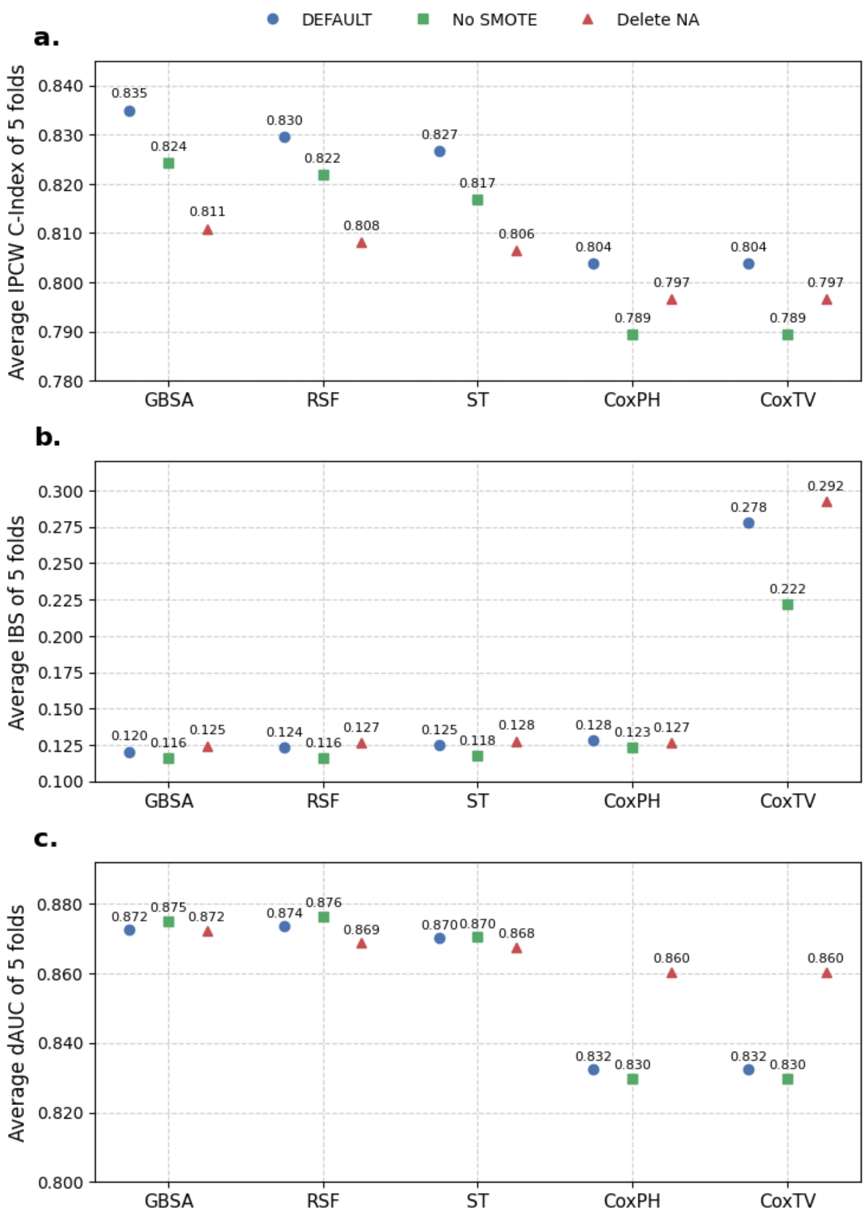

Supplementary Fig S5 Comparison of model performance across different data preprocessing strategies. (a) IPCW C-index Comparison; (b) IBS Comparison; (c) dAUC Comparison

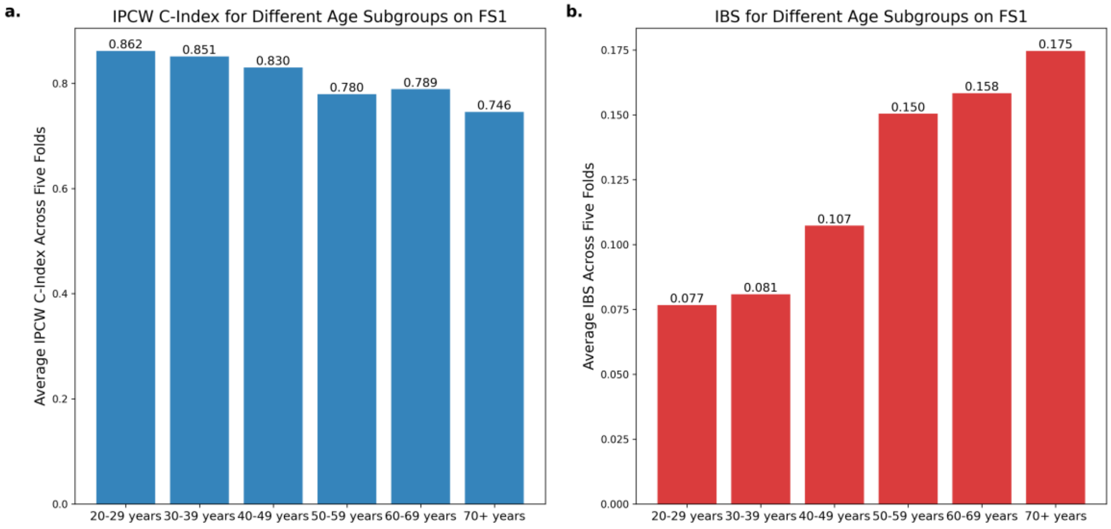

Supplementary Fig S6 Performance of the GBSA Model Across Age-Specific Subgroups Using FS1 (a) IPCW C-Index by Age Group Averaged Over Fivefold Cross-Validation; (b) IBS by Age Group Averaged Over Fivefold Cross-Validation

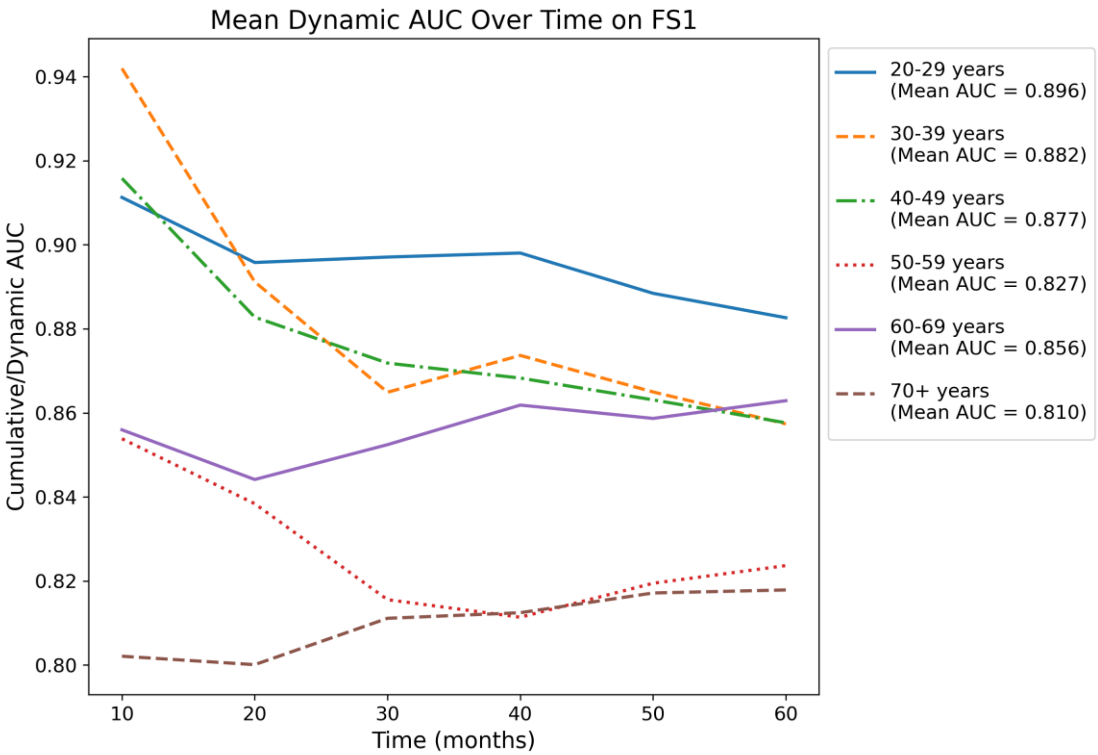

Supplementary Fig S7 dAUC Curves of the GBSA Model Across Age Groups Using FS1

		Fold1			Fold2			Fold3			Fold4			Fold5		
Variable	N	Train,N=7,268[1]	Test,N=1,817[1]	p-value	Train,N=7,268[1]	Test,N=1,817[1]	p-value	Train,N=7,268[1]	Test,N=1,817[1]	p-value	Train,N=7,268[1]	Test,N=1,817[1]	p-value	Train,N=7,268[1]	Test,N=1,817[1]	p-value
Age	9,085			0.132			0.222			0.802			0.442			0.662
20-29years		462(6.4%)	114(6.3%)		469(6.5%)	107(5.9%)		460(6.3%)	116(6.4%)		463(6.4%)	113(6.2%)		450(6.2%)	126(6.9%)	
30-39years		1,667(23%)	466(26%)		1,727(24%)	406(22%)		1,706(23%)	427(24%)		1,727(24%)	406(22%)		1,705(23%)	428(24%)	
40-49years		2,001(28%)	501(28%)		1,963(27%)	539(30%)		2,023(28%)	479(26%)		2,006(28%)	496(27%)		2,015(28%)	487(27%)	
50-59years		1,539(21%)	360(20%)		1,537(21%)	362(20%)		1,512(21%)	387(21%)		1,503(21%)	396(22%)		1,505(21%)	394(22%)	
60-69years		924(13%)	204(11%)		901(12%)	227(12%)		889(12%)	239(13%)		883(12%)	245(13%)		915(13%)	213(12%)	
70+years		675(9.3%)	172(9.5%)		671(9.2%)	176(9.7%)		678(9.3%)	169(9.3%)		686(9.4%)	161(8.9%)		678(9.3%)	169(9.3%)	
Tumor_Stage	9,085			0.552			0.832			0.922			0.612			0.972
Unknown/unstaged		235(3.2%)	62(3.4%)		233(3.2%)	64(3.5%)		237(3.3%)	60(3.3%)		244(3.4%)	53(2.9%)		239(3.3%)	58(3.2%)	
Localized		3,646(50%)	914(50%)		3,639(50%)	921(51%)		3,658(50%)	902(50%)		3,658(50%)	902(50%)		3,639(50%)	921(51%)	
Regional		2,462(34%)	591(33%)		2,450(34%)	603(33%)		2,430(33%)	623(34%)		2,423(33%)	630(35%)		2,447(34%)	606(33%)	
Distant		925(13%)	250(14%)		946(13%)	229(13%)		943(13%)	232(13%)		943(13%)	232(13%)		943(13%)	232(13%)	
Differentiation	9,085			0.152			0.152			0.212			0.292			0.522
GradeI(Welldifferentiated)		763(10%)	217(12%)		809(11%)	171(9.4%)		771(11%)	209(12%)		790(11%)	190(10%)		787(11%)	193(11%)	
GradeII(Moderatelydifferentiated)		4,444(61%)	1,076(59%)		4,383(60%)	1,137(63%)		4,397(60%)	1,123(62%)		4,438(61%)	1,082(60%)		4,418(61%)	1,102(61%)	
GradeIII(Poorlydifferentiated)		1,885(26%)	488(27%)		1,906(26%)	467(26%)		1,932(27%)	441(24%)		1,867(26%)	506(28%)		1,902(26%)	471(26%)	
GradeIV(Undifferentiated/anaplastic)		176(2.4%)	36(2.0%)		170(2.3%)	42(2.3%)		168(2.3%)	44(2.4%)		173(2.4%)	39(2.1%)		161(2.2%)	51(2.8%)	
Tumor_Size	9,085			0.902			0.532			0.692			0.050			0.452
other		229(3.2%)	61(3.4%)		225(3.1%)	65(3.6%)		232(3.2%)	58(3.2%)		248(3.4%)	42(2.3%)		226(3.1%)	64(3.5%)	
<=40mm		4,977(68%)	1,240(68%)		4,986(69%)	1,231(68%)		4,959(68%)	1,258(69%)		4,953(68%)	1,264(70%)		4,993(69%)	1,224(67%)	
>40mm		2,062(28%)	516(28%)		2,057(28%)	521(29%)		2,077(29%)	501(28%)		2,067(28%)	511(28%)		2,049(28%)	529(29%)	
InSitu_Malignant_Cancer_Count	9,085			0.162			0.322			0.612			0.312			0.362
=1		6,356(87%)	1,611(89%)		6,386(88%)	1,581(87%)		6,380(88%)	1,587(87%)		6,361(88%)	1,606(88%)		6,385(88%)	1,582(87%)	
>1		912(13%)	206(11%)		882(12%)	236(13%)		888(12%)	230(13%)		907(12%)	211(12%)		883(12%)	235(13%)	
Chemotherapy_Received	9,085			0.322			0.922			0.432			0.872			0.792
No		3,917(54%)	1,003(55%)		3,938(54%)	982(54%)		3,951(54%)	969(53%)		3,933(54%)	987(54%)		3,941(54%)	979(54%)	
Yes		3,351(46%)	814(45%)		3,330(46%)	835(46%)		3,317(46%)	848(47%)		3,335(46%)	830(46%)		3,327(46%)	838(46%)	
Histology_Adenocarcinoma	9,085			0.722			0.662			0.582			0.512			>0.99[2]
No		5,459(75%)	1,372(76%)		5,472(75%)	1,359(75%)		5,474(75%)	1,357(75%)		5,454(75%)	1,377(76%)		5,465(75%)	1,366(75%)	
Yes		1,809(25%)	445(24%)		1,796(25%)	458(25%)		1,794(25%)	460(25%)		1,814(25%)	440(24%)		1,803(25%)	451(25%)	
Histology_Adenosquamous_Carcinoma	9,085			0.882			0.652			0.652			0.292			0.222
No		7,045(97%)	1,760(97%)		7,041(97%)	1,764(97%)		7,047(97%)	1,758(97%)		7,051(97%)	1,754(97%)		7,036(97%)	1,769(97%)	

Yes		223(3.1%)	57(3.1%)		227(3.1%)	53(2.9%)		221(3.0%)	59(3.2%)		217(3.0%)	63(3.5%)		232(3.2%)	48(2.6%)	
Histology_Neuroendocrine_Carcinoma	9,085			0.632			0.582			0.132			0.842			0.222
No		7,227(99%)	1,805(99%)		7,224(99%)	1,808(100%)		7,230(99%)	1,802(99%)		7,225(99%)	1,807(99%)		7,222(99%)	1,810(100%)	
Yes		41(0.6%)	12(0.7%)		44(0.6%)	9(0.5%)		38(0.5%)	15(0.8%)		43(0.6%)	10(0.6%)		46(0.6%)	7(0.4%)	
Histology_Squamous_Cell_Carcinoma	9,085			0.392			0.302			0.922			0.472			0.422
No		2,687(37%)	652(36%)		2,652(36%)	687(38%)		2,673(37%)	666(37%)		2,658(37%)	681(37%)		2,686(37%)	653(36%)	
Yes		4,581(63%)	1,165(64%)		4,616(64%)	1,130(62%)		4,595(63%)	1,151(63%)		4,610(63%)	1,136(63%)		4,582(63%)	1,164(64%)	
Marital_Status_Divorced	9,085			0.272			0.532			0.342			0.752			0.082
No		6,370(88%)	1,575(87%)		6,348(87%)	1,597(88%)		6,368(88%)	1,577(87%)		6,360(88%)	1,585(87%)		6,334(87%)	1,611(89%)	
Yes		898(12%)	242(13%)		920(13%)	220(12%)		900(12%)	240(13%)		908(12%)	232(13%)		934(13%)	206(11%)	
Marital_Status_Married	9,085			0.532			0.028			0.962			0.102			>0.99[2]
No		3,544(49%)	901(50%)		3,598(50%)	847(47%)		3,557(49%)	888(49%)		3,525(49%)	920(51%)		3,556(49%)	889(49%)	
Yes		3,724(51%)	916(50%)		3,670(50%)	970(53%)		3,711(51%)	929(51%)		3,743(51%)	897(49%)		3,712(51%)	928(51%)	
Marital_Status_Separated	9,085			0.532			0.972			0.882			0.312			0.132
No		7,132(98%)	1,787(98%)		7,135(98%)	1,784(98%)		7,136(98%)	1,783(98%)		7,130(98%)	1,789(98%)		7,143(98%)	1,776(98%)	
Yes		136(1.9%)	30(1.7%)		133(1.8%)	33(1.8%)		132(1.8%)	34(1.9%)		138(1.9%)	28(1.5%)		125(1.7%)	41(2.3%)	
Marital_Status_Single	9,085			0.462			0.071			0.152			0.036			0.662
No		5,318(73%)	1,314(72%)		5,275(73%)	1,357(75%)		5,281(73%)	1,351(74%)		5,341(73%)	1,291(71%)		5,313(73%)	1,319(73%)	
Yes		1,950(27%)	503(28%)		1,993(27%)	460(25%)		1,987(27%)	466(26%)		1,927(27%)	526(29%)		1,955(27%)	498(27%)	
Marital_Status_Widowed	9,085			0.182			0.902			0.432			0.742			0.322
No		6,734(93%)	1,700(94%)		6,746(93%)	1,688(93%)		6,755(93%)	1,679(92%)		6,744(93%)	1,690(93%)		6,757(93%)	1,677(92%)	
Yes		534(7.3%)	117(6.4%)		522(7.2%)	129(7.1%)		513(7.1%)	138(7.6%)		524(7.2%)	127(7.0%)		511(7.0%)	140(7.7%)	
Primary_Site_Surgery_Destruction	9,085			0.812			0.402			0.722			0.342			0.812
No		7,212(99%)	1,802(99%)		7,214(99%)	1,800(99%)		7,210(99%)	1,804(99%)		7,208(99%)	1,806(99%)		7,212(99%)	1,802(99%)	
Yes		56(0.8%)	15(0.8%)		54(0.7%)	17(0.9%)		58(0.8%)	13(0.7%)		60(0.8%)	11(0.6%)		56(0.8%)	15(0.8%)	
Primary_Site_Surgery_None	9,085			0.802			0.722			0.612			0.612			0.682
No		4,487(62%)	1,116(61%)		4,489(62%)	1,114(61%)		4,473(62%)	1,130(62%)		4,473(62%)	1,130(62%)		4,490(62%)	1,113(61%)	
Yes		2,781(38%)	701(39%)		2,779(38%)	703(39%)		2,795(38%)	687(38%)		2,795(38%)	687(38%)		2,778(38%)	704(39%)	
Primary_Site_Surgery_Resection	9,085			0.742			0.662			0.762			0.462			0.782
No		2,865(39%)	724(40%)		2,863(39%)	726(40%)		2,877(40%)	712(39%)		2,885(40%)	704(39%)		2,866(39%)	723(40%)	
Yes		4,403(61%)	1,093(60%)		4,405(61%)	1,091(60%)		4,391(60%)	1,105(61%)		4,383(60%)	1,113(61%)		4,402(61%)	1,094(60%)	
Primary_Site_Cervix_Uteri	9,085			0.085			0.102			0.852			0.812			0.902
No		1,760(24%)	405(22%)		1,705(23%)	460(25%)		1,729(24%)	436(24%)		1,736(24%)	429(24%)		1,730(24%)	435(24%)	
Yes		5,508(76%)	1,412(78%)		5,563(77%)	1,357(75%)		5,539(76%)	1,381(76%)		5,532(76%)	1,388(76%)		5,538(76%)	1,382(76%)	

Primary_Site_Endocervix	9,085			0.252			0.112			0.822			0.882			0.412
No		5,780(80%)	1,467(81%)		5,822(80%)	1,425(78%)		5,801(80%)	1,446(80%)		5,800(80%)	1,447(80%)		5,785(80%)	1,462(80%)	
Yes		1,488(20%)	350(19%)		1,446(20%)	392(22%)		1,467(20%)	371(20%)		1,468(20%)	370(20%)		1,483(20%)	355(20%)	
Primary_Site_Exocervix	9,085			0.402			0.802			0.632			0.772			0.172
No		7,113(98%)	1,784(98%)		7,119(98%)	1,778(98%)		7,115(98%)	1,782(98%)		7,116(98%)	1,781(98%)		7,125(98%)	1,772(98%)	
Yes		155(2.1%)	33(1.8%)		149(2.1%)	39(2.1%)		153(2.1%)	35(1.9%)		152(2.1%)	36(2.0%)		143(2.0%)	45(2.5%)	
Race_Black	9,085			0.852			0.382			0.162			0.040			0.682
No		6,513(90%)	1,631(90%)		6,505(90%)	1,639(90%)		6,499(89%)	1,645(91%)		6,539(90%)	1,605(88%)		6,520(90%)	1,624(89%)	
Yes		755(10%)	186(10%)		763(10%)	178(9.8%)		769(11%)	172(9.5%)		729(10%)	212(12%)		748(10%)	193(11%)	
Race_White	9,085			0.492			0.692			0.056			0.242			0.652
No		1,807(25%)	466(26%)		1,825(25%)	448(25%)		1,850(25%)	423(23%)		1,799(25%)	474(26%)		1,811(25%)	462(25%)	
Yes		5,461(75%)	1,351(74%)		5,443(75%)	1,369(75%)		5,418(75%)	1,394(77%)		5,469(75%)	1,343(74%)		5,457(75%)	1,355(75%)	
Radiotherapy_Received	9,085			0.082			0.802			0.302			0.802			0.842
No		3,522(48%)	922(51%)		3,560(49%)	884(49%)		3,575(49%)	869(48%)		3,560(49%)	884(49%)		3,559(49%)	885(49%)	
Yes		3,746(52%)	895(49%)		3,708(51%)	933(51%)		3,693(51%)	948(52%)		3,708(51%)	933(51%)		3,709(51%)	932(51%)	
Lymph_Node_Surgery_Extent_Removed	9,085			0.672			>0.99[2]			0.512			0.782			0.602
No		4,459(61%)	1,105(61%)		4,451(61%)	1,113(61%)		4,439(61%)	1,125(62%)		4,446(61%)	1,118(62%)		4,461(61%)	1,103(61%)	
Yes		2,809(39%)	712(39%)		2,817(39%)	704(39%)		2,829(39%)	692(38%)		2,822(39%)	699(38%)		2,807(39%)	714(39%)	
Lymph_Node_Surgery_Extent_None_Removed	9,085			0.622			>0.99[2]			0.762			0.842			>0.99[2]
No		2,942(40%)	747(41%)		2,951(41%)	738(41%)		2,957(41%)	732(40%)		2,955(41%)	734(40%)		2,951(41%)	738(41%)	
Yes		4,326(60%)	1,070(59%)		4,317(59%)	1,079(59%)		4,311(59%)	1,085(60%)		4,313(59%)	1,083(60%)		4,317(59%)	1,079(59%)	
Lymph_Node_Surgery_Extent_Sentinel_Biopsy	9,085			0.882			0.562			0.051			0.882			0.102
No		7,230(99%)	1,807(99%)		7,228(99%)	1,809(100%)		7,235(100%)	1,802(99%)		7,230(99%)	1,807(99%)		7,225(99%)	1,812(100%)	
Yes		38(0.5%)	10(0.6%)		40(0.6%)	8(0.4%)		33(0.5%)	15(0.8%)		38(0.5%)	10(0.6%)		43(0.6%)	5(0.3%)	
time	9,085	60.000(28.000,60.000)	60.000(28.000,60.000)	0.623	60.000(28.000,60.000)	60.000(28.000,60.000)	0.663	60.000(28.000,60.000)	60.000(31.000,60.000)	0.333	60.000(28.000,60.000)	60.000(28.000,60.000)	0.563	60.000(28.750,60.000)	60.000(27.000,60.000)	0.523
event	9,085			>0.99[2]			>0.99[2]			>0.99[2]			>0.99[2]			>0.99[2]
No		5,196(71%)	1,299(71%)		5,196(71%)	1,299(71%)		5,196(71%)	1,299(71%)		5,196(71%)	1,299(71%)		5,196(71%)	1,299(71%)	
Yes		2,072(29%)	518(29%)		2,072(29%)	518(29%)		2,072(29%)	518(29%)		2,072(29%)	518(29%)		2,072(29%)	518(29%)	

1n(%);Median(IQR)

2Pearson'sChi-squaredtest

3Wilcoxonranksumtest

Supplementary Table S1 Baseline Characteristics of Participants Across Five Folds

Variable	Fold 1			Fold 2			Fold 3			Fold 4			Fold 5		
	Chi-Squared	p-value	-log2(p-value)	Chi-Squared	p-value	-log2(p-value)	Chi-Squared	p-value	-log2(p-value)	Chi-Squared	p-value	-log2(p-value)	Chi-Squared	p-value	-log2(p-value)
**Age**	2.27	0.13	2.92	9.56	<0.005	8.98	3.13	0.08	3.7	0.09	0.76	0.4	1.3	0.25	1.98
**Chemotherapy_Received**	202.53	<0.005	150.26	163.63	<0.005	122.04	156.41	<0.005	116.81	169.41	<0.005	126.24	156.63	<0.005	116.97
**Differentiation**	1.72	0.19	2.39	0.06	0.8	0.32	0.9	0.34	1.54	0.01	0.91	0.13	0.3	0.58	0.77
**Histology_Adenocarcinoma**	6.63	0.01	6.64	18.46	<0.005	15.81	12.88	<0.005	11.56	8.62	<0.005	8.23	6.25	0.01	6.33
**Histology_Adenosquamous_Carcinoma**	0.2	0.66	0.61	0	0.96	0.05	0	0.98	0.03	0	0.98	0.03	0.04	0.85	0.23
**Histology_Neuroendocrine_Carcinoma**	2.99	0.08	3.58	2.42	0.12	3.06	1.02	0.31	1.68	4.44	0.04	4.83	3.84	0.05	4.32
**Histology_Squamous_Cell_Carcinoma**	2.54	0.11	3.18	9.4	<0.005	8.85	10.36	<0.005	9.6	1.11	0.29	1.78	3.01	0.08	3.6
**InSitu_Malignant_Cancer_Count**	8.52	<0.005	8.15	2.01	0.16	2.68	1.46	0.23	2.14	11.59	<0.005	10.56	8.57	<0.005	8.19
**Lymph_Node_Surgery_Extent_None_Removed**	9.38	<0.005	8.84	28.36	<0.005	23.25	11.08	<0.005	10.16	30.09	<0.005	24.53	11.93	<0.005	10.83
**Lymph_Node_Surgery_Extent_Removed**	2.58	0.11	3.21	6.29	0.01	6.37	0.56	0.46	1.13	11.59	<0.005	10.56	4.33	0.04	4.74
**Lymph_Node_Surgery_Extent_Sentinel_Biopsy**	0.16	0.69	0.53	2.51	0.11	3.14	0.75	0.38	1.38	1.14	0.29	1.81	5.6	0.02	5.8
**Marital_Status_Divorced**	80.97	<0.005	61.92	43.62	<0.005	34.55	66.63	<0.005	51.44	80.9	<0.005	61.87	28.25	<0.005	23.16
**Marital_Status_Married**	125.87	<0.005	94.62	95.73	<0.005	72.69	109.45	<0.005	82.68	130.62	<0.005	98.07	53.24	<0.005	41.62
**Marital_Status_Separated**	31.17	<0.005	25.33	22.91	<0.005	19.16	20.62	<0.005	17.45	32.61	<0.005	26.4	21.21	<0.005	17.89
**Marital_Status_Single**	148.11	<0.005	110.78	112.62	<0.005	84.99	145.21	<0.005	108.67	146.19	<0.005	109.38	110.79	<0.005	83.65
**Marital_Status_Widowed**	174.29	<0.005	129.78	93.68	<0.005	71.19	114.65	<0.005	86.46	151.88	<0.005	113.51	117.11	<0.005	88.25
**Primary_Site_Cervix_Uteri**	35.71	<0.005	28.7	25.64	<0.005	21.21	48.42	<0.005	38.08	51.4	<0.005	40.27	28.6	<0.005	23.42
**Primary_Site_Endocervix**	3.84	0.05	4.32	8.6	<0.005	8.22	23.38	<0.005	19.52	18.63	<0.005	15.94	9.12	<0.005	8.63
**Primary_Site_Exocervix**	1.87	0.17	2.55	1.47	0.22	2.15	1.08	0.3	1.74	4.01	0.05	4.47	0.03	0.87	0.2
**Primary_Site_Surgery_Destruction**	1.66	0.2	2.34	1.92	0.17	2.6	4.76	0.03	5.1	5.8	0.02	5.97	3.64	0.06	4.15
**Primary_Site_Surgery_None**	54.38	<0.005	42.46	11.7	<0.005	10.64	19.45	<0.005	16.56	24.74	<0.005	20.54	60.89	<0.005	47.23
**Primary_Site_Surgery_Resection**	0.26	0.61	0.71	7.19	0.01	7.09	1.22	0.27	1.89	9.65	<0.005	9.05	0.01	0.93	0.1
**Race_Black**	27.1	<0.005	22.31	4.19	0.04	4.62	22.79	<0.005	19.08	12.83	<0.005	11.51	13.82	<0.005	12.28
**Race_White**	29.28	<0.005	23.93	5.77	0.02	5.94	46.75	<0.005	36.85	27.62	<0.005	22.69	17.15	<0.005	14.82

**Radiotherapy_Received**	59.36	<0.005	46.12	87.57	<0.005	66.73	84.55	<0.005	64.54	123.54	<0.005	92.93	112.56	<0.005	84.94
**Tumor_Size**	7.02	0.01	6.95	0.24	0.63	0.67	0.26	0.61	0.71	0.59	0.44	1.18	3.34	0.07	3.89
**Tumor_Stage**	75.6	<0.005	58	126.44	<0.005	95.04	63.23	<0.005	48.95	75.36	<0.005	57.82	111.45	<0.005	84.14

Supplementary Table S2 Proportional Hazards Assumption Test Results

Study	DOI	Cancer Type	Survival Type	Dataset	Models	Interpretability Methods	Key Findings
Our Study	N/A	CC	CSS	SEER	CoxPH, CoxTV, GBSA, RSF, ST	SHAP values	GBSA outperformed other models (IPCW C-index: 0.835, IBS: 0.120). SHAP identified tumor stage and surgery as key predictors.
Comparative study of machine learning and statistical survival models for enhancing cervical cancer prognosis and risk factor assessment using SEER data	10.1038/s41598-024-72790-5	CC	OS	SEER	RSF, CoxPH	None	RSF outperformed CoxPH for OS when the PH assumption was violated.
A 5-year survival status prognosis of nonmetastatic cervical cancer patients through machine learning algorithms	10.1002/cam4.5477	Non-Metastatic CC	OS	SEER	Logistic Regression, Random Forest, Support Vector Machine, Extreme Gradient Boosting	None	ML algorithms improved OS prediction, but lacked cross-validation.
Oral cancer prognosis based on clinicopathologic and genomic markers using a hybrid of feature selection and machine learning methods	10.1186/1471-2105-14-170	Oral Cancer	OS	Single-center data	Hybrid feature selection, ML	None	Applied ML to oral cancer prognosis but did not use interpretability techniques like SHAP.
Cervical cancer survival prediction by machine learning algorithms: a systematic review	10.1186/s12885-023-10808-3	CC	OS	Literature review	Various ML algorithms	Varies by study	Systematic review on ML models for OS prediction in CC, highlighting the need for CSS-specific studies.

Supplementary Table S3 Comparative Analysis of Survival Models
